# Myxovirus Resistance A Protein Expression in Idiopathic Inflammatory Myopathies and Hereditary Muscle Diseases with Inflammatory Cell Infiltration: A North African Study

**DOI:** 10.3390/ijms27073091

**Published:** 2026-03-28

**Authors:** Emna Farhat, Imen Zamali, Thouraya Ben Younes, Hedia Klaa, Werner Stenzel, Samar Samoud, Hanen Ben Rhouma, Yousr Galai, Ilhem Ben Youssef-Turki, Ichraf Kraoua, Mélika Ben Ahmed, Ahlem Ben Hmid

**Affiliations:** 1 Faculty of Medicine of Tunis, Tunis El Manar University, Tunis 1007, Tunisia; imen.zamali@fmt.utm.tn (I.Z.); thouraya.benyounes@fmt.utm.tn (T.B.Y.); hedia.klaa@fmt.utm.tn (H.K.); hanene.benrhouma@fmt.utm.tn (H.B.R.); ilhem.byt@fmt.utm.tn (I.B.Y.-T.); ichraf.kraoua@fmt.utm.tn (I.K.); melika.benahmed@fmt.utm.tn (M.B.A.); ahlem.benhmid@fmt.utm.tn (A.B.H.); 2 Laboratory of the Transmission, Control and Immunobiology of Infections, Pasteur Institute, Tunis 1002, Tunisia; samar.samoud@pasteur.tn; 3 Department of Clinical Immunology, Pasteur Institute of Tunis, Tunis 1002, Tunisia; yousr.galai@pasteur.tn; 4 Department of Paediatric Neurology, National Institute Mongi Ben Hamida of Neurology, Tunis 1007, Tunisia; 5 Department of Neuropathology, Charité Medicine University, 10117 Berlin, Germany; werner.stenzel@charite.de

**Keywords:** inflammatory myopathies, muscular dystrophies, myxovirus resistance A, type I interferon, muscle biopsy, immunohistochemistry, immune system

## Abstract

Muscle biopsy (MB) is an important tool to help differentiate idiopathic inflammatory myopathies (IIMs) from hereditary muscular diseases (HMDs). The usefulness of immunohistochemical stains of the major histocompatibility complex class I and the membrane attack complex are controversial, as both may be identified in some HMDs. More sensitive markers of IIMs have recently been used, such as myxovirus resistance A (MxA), a type I interferon-inducible protein. We selected skeletal MB samples from 81 patients diagnosed with IIM and HMD harbouring overt inflammatory infiltrates on their MBs in the period between March 2022 and September 2024. Two groups were identified: the IIM group (46 cases) and the HMD group (35 cases). We characterized and compared the patterns of MxA protein expression among the two groups. In the IIM group, positive sarcoplasmic MxA expression was detected on the myofibres of 10 patients (24%), among whom were eight dermatomyositis patients. In the HMD group, we did not identify any sarcoplasmic positivity. However, five patients (14%) showed positive labelling restricted to the sarcolemmal membrane, including non-necrotic or regenerating fibres. Our study demonstrates the value of MxA for increasing dermatomyositis diagnostic accuracy and suggests the potential role of interferon type I in the pathophysiology of HMD.

## 1. Introduction

Idiopathic inflammatory myopathies (IIMs) are a heterogeneous group of autoimmune muscle diseases. Diagnosis of IIM requires a multidisciplinary approach, composed of a clinical evaluation, autoantibody profiling, and myo-pathological assessment. Classification of IIM has been based on clinical, serological and morphological features that have led to four major subsets: inclusion body myositis (IBM), dermatomyositis (DM), immune-mediated necrotizing myopathy (IMNM) and antisynthetase syndrome (ASyS) [1,2]. Furthermore, non-specific myositis is associated with various autoimmune diseases. Overlap myositis (OM) is defined by the presence of myositis and at least one clinical overlap feature with those present in connective tissue diseases and/or an overlap antibody [3]. The subgroup of OM patients displaying systemic sclerosis has been denominated as scleromyositis [4]. Hereditary muscle diseases (HMDs) are a heterogeneous family of genetic diseases involving muscle tissue, characterized by progressive muscle wasting and weakness of variable distribution and severity. They mainly include the following groups: congenital myopathies; metabolic myopathies; and muscular dystrophies (MDs), including congenital muscular dystrophies (CMDs), limb-girdle muscular dystrophies (LGMDs) and facioscapulohumeral MDs [5].

A muscle biopsy (MB) remains the gold standard for diagnosing IIM and HMD, although genetic testing plays an essential and undisputed role in the diagnosis of inherited muscle diseases. Morphologically, dystrophic muscle is typically characterized by necrotic and regenerating myofibres, replacement of muscle tissue with fatty and/or fibrotic tissue, and occasionally cellular infiltration [5]. IIMs share several hallmark pathological features, notably muscle fibre necrosis and regeneration, along with the presence of inflammatory cells, many of which are involved in tissue repair processes, constituting the key indicators of IIM [6]. However, conventional light microscopy may not reliably distinguish between IIM and HMD. On one hand, in certain IIM subtypes such as IMNM, the muscle morphology may show minimal or no significant inflammatory infiltrate [7]. On the other hand, pronounced inflammatory cell infiltration may also be seen in various HMDs, potentially leading to the misdiagnosis of IIM and the administration of inappropriate immunosuppressive therapies [8,9]. Dysferlinopathies, calpainopathies, and facioscapulohumeral MD are among the HMDs most commonly mistaken for IIM, as they often exhibit prominent inflammatory infiltrates on MBs that closely resemble those seen in IIM [10,11]. In such cases, immunohistochemistry (IHC) is particularly valuable for guiding accurate diagnosis.

Immunological markers, particularly major histocompatibility complex class I (MHC-I) and the membrane attack complex (MAC), have been employed for decades to help avoid morphological misinterpretations, and are now routinely used in the pathological diagnosis of myopathies [12]. However, the diagnostic value of these immunohistochemical markers has become increasingly debated. While several studies have highlighted the high sensitivity of MHC-I expression for differentiating IIM from HMD [7,13], other reports have raised concerns about its low specificity. Indeed, MHC-I can also be strongly expressed in certain HMDs, particularly in various forms of MD [14,15,16,17]. Moreover, in some IIM cases, especially those lacking inflammatory infiltrates or showing minimal histopathological changes, MHC-I expression may be absent altogether. These limitations have made the diagnostic utility of MHC-I expression increasingly controversial, prompting pathologists to search for more specific and reliable markers. Over the past decade, significant advances have been made in understanding the role of type I interferon (IFN-I) signalling in the pathogenesis of DM. In particular, IFN-I-inducible genes, such as interferon-stimulated gene 15 (ISG15) and myxovirus resistance protein A (MxA), have been shown to be upregulated in DM [18,19].

Importantly, the MxA IFN-I-inducible protein has been consistently detected by IHC in the perifascicular regions of MBs from DM patients. MxA expression in the cytoplasm of myofibres has demonstrated higher sensitivity than perifascicular atrophy (PFA) or capillary MAC deposition, and is now considered the most specific immunopathological marker for DM [20,21]. Although not completely elucidated, the role of IFN in the physiopathology of IIM has been increasingly scrutinized, especially in juvenile and also in adult DM [22,23,24]. In contrast, MxA expression in myofibres is rarely observed in other forms of IIM and, to our knowledge, has never been reported in HMD. In this study, we aimed to characterize the types and distribution patterns of MxA protein expression across the various subtypes of IIM and HMD, in order to assess its diagnostic utility and specificity.

## 2. Results

### 2.1. Patient Characteristics

#### 2.1.1. Clinical Characteristics of the IIM Group

The first group included 46 patients (57% of the cohort) who fulfilled both the clinical and histopathological criteria for IIM based on the ENMC classification. The distribution of IIM subtypes was as follows: OM: 54% (*n* = 25); DM: 22% (*n* = 10); ASyS: 9% (*n* = 4); IBM: 4% (*n* = 2); and IMNM: 11% (*n* = 5). The clinical features are summarized in Table 1. Most of the patients were adults (87%, *n* = 40), with a predominance of the female sex (sex ratio: 36/46). Only six patients were classified as having JM, with three confirmed juvenile DM. A personal and/or familial history of autoimmune diseases was reported in 70% of all the patients (Table 1).

The OM subgroup was the most prevalent among the IIM patients, comprising 54% (*n* = 25) of the group. Based on the clinical features and MAAs, the OM cases were classified into: scleromyositis: *n* = 2 (4%); and non-specific OM: *n* = 23 (50%). Within the non-specific OM group, secondary autoimmune diseases were frequently identified, most notably systemic lupus erythematosus (SLE) (*n* = 3) and Sjögren’s syndrome (*n* = 4). ILD was diagnosed in one scleromyositis patient.

The DM subgroup included 10 patients (22% of IIM cases), with seven adults and three juvenile DM. Among them, 80% presented with typical DM-specific cutaneous manifestations, including Gottron’s papules and a heliotrope rash. The most commonly detected MSAs in the DM subgroup were: anti-MDA5 (*n* = 3), anti-NXP2 (*n* = 3), anti-TIF1γ (*n* = 1) and anti-Mi2 (*n* = 1). Two DM patients were MSA-negative, though all tested positive for anti-nuclear antibodies. In one case, anti-Ku and anti-Ro52 antibodies were co-detected. No cases with anti-SAE antibodies were found. All the patients positive for anti-MDA5 antibodies had severe muscle weakness, and one developed rapidly progressive ILD. Additionally, two adult female DM patients were diagnosed with ovarian cancer, one preceding and one following the onset of DM symptoms.

The ASyS subgroup included four adult patients (9%): one patient was positive for anti-Jo1 antibodies, and one patient had positive anti-PL7 antibodies. Two ASyS patients had swallowing and respiratory difficulties at the time of the MB, contrasting with normal CK levels. ILD was diagnosed in three cases. The IMNM subgroup consisted of five patients (11%). Their autoantibody profiles were as follows: anti-SRP (*n* = 2), anti-HMGCR (*n* = 1), and seronegative (*n* = 2). The two seronegative patients had notable clinical histories of type 2 diabetes mellitus and had been on long-term statin therapy for dyslipidaemia (≥2 years), supporting a statin-associated IMNM phenotype.

#### 2.1.2. Clinical Characteristics of the HMD Group

Thirty-five patients (43%) were diagnosed with HMD based on their clinical phenotypes, muscle pathology, sarcolemmal protein IHC data, and genetic studies. All the patients in this group presented with elevated serum CK levels and exhibited active myopathic patterns on a needle electromyography. The majority of patients were young, with 60% (*n* = 21) under the age of 18 years, and the group had a male predominance (54%, *n* = 19). The cohort included patients diagnosed with different subtypes of HMDs. The demographic and clinical data of the HMD group are illustrated in Table 2.

The metabolic myopathy case was included in this cohort as the MB was performed during an episode of rhabdomyolysis, which revealed significant inflammatory infiltrate.

All the patients in this group exhibited active myopathic patterns on the needle electromyography and presented with elevated serum CK levels.

### 2.2. Muscle Pathology and MxA Protein Expression

**Figure 1:**
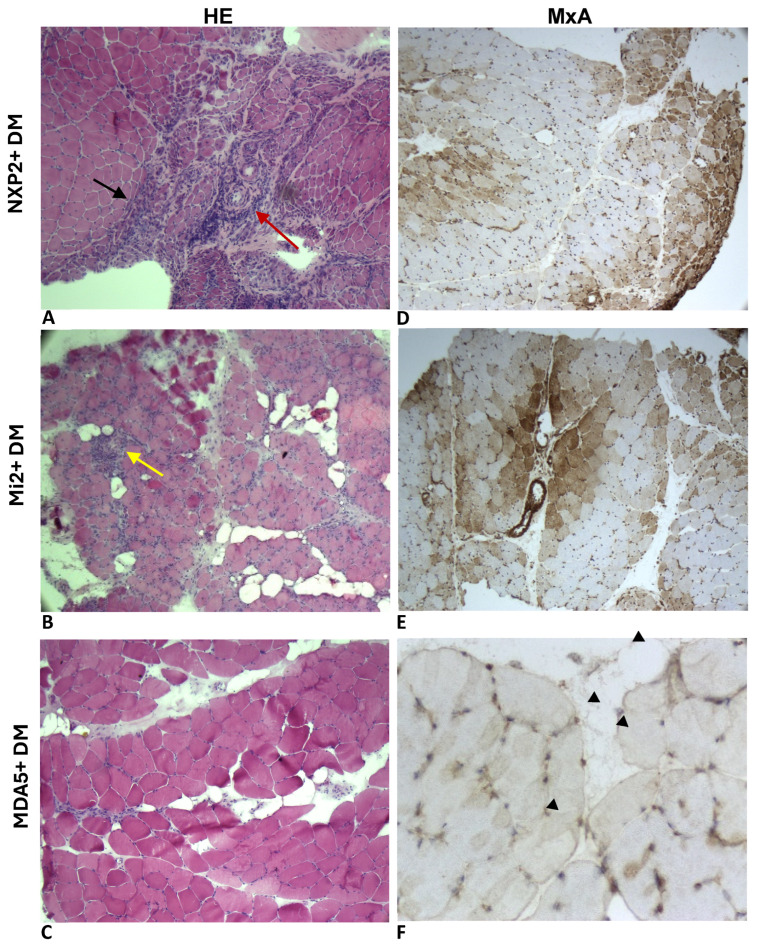
Different patterns of pathology and MxA immunohistochemical staining of autoantibody-related DM subgroups: (**A**) Juvenile NXP2 + DM with perifascicular atrophy (black arrow) and perivascular inflammatory infiltrate (red arrow). (**B**) Mi2 + DM with panfascicular necrosis and inflammatory infiltrate (yellow arrow). (**C**) MDA5 + DM with mild myogenic pattern. (**D**) Perifascicular positive sarcoplasmic expression of MxA. (**E**) Scattered positive sarcoplasmic expression of MxA. (**F**) Perifascicular sarcolemmal expression of MxA (triangles) with no apparent sarcoplasmic MxA expression). Images (**A**,**B**,**D**): magnification 10×; images (**C**,**E**): magnification 40×; image (**F**): magnification 100×. DM: dermatomyositis.

In our cohort, 16 patients (19%) demonstrated positive staining for anti-MxA antibodies in their myofibres, with the staining patterns observed either in the sarcoplasm (10 cases) or the sarcolemma (six cases). Notably, all the patients with sarcoplasmic MxA expression were from the IIM group, while sarcolemmal MxA positivity was predominantly seen in the HMD group (five cases), *p* = 0.001 (Table 3). Additionally, one anti-MDA5-positive DM patient displayed this unique pattern in the perifascicular region (Figure 1F).

#### 2.2.1. Group 1: Idiopathic Inflammatory Myopathies

On the conventional light microscopy, all the IIM patients showed clear inflammatory cell infiltrate as well as necrosis and regeneration of muscle fibres. We observed variable patterns depending on the IIM subtype: non-specific myogenic changes in OM, perivascular inflammatory infiltrate with PFA in DM, severe atrophy with nuclear inclusions and rimmed vacuoles in IBM, fibrosis and vasculopathy in scleromyositis, perifascicular necrosis and inflammation in ASyS, and diffuse necrotizing myopathy with moderate inflammatory infiltrate in IMNM (Figure 1A–F).

Sarcoplasmic MxA expression was identified in 10 IIM patients (22%), with the majority being from the DM group (eight cases), and two cases of non-specific OM. Of those expressing sarcoplasmic MxA, four patients (three DM, one OM) showed a perifascicular pattern, while six cases exhibited a diffuse and scattered pattern. MxA reactivity was most prominent in the DM group (80%), with variability observed among the autoantibody-related subgroups: the anti-NXP2- and anti-TIF1-γ-positive DM patients showed intense sarcoplasmic MxA staining. The anti-MDA5-positive DM patients exhibited negative-to-moderate MxA staining. The distribution patterns also differed: the anti-MDA5-positive DM patients showed a scattered or diffuse MxA distribution. Interestingly, a 50-year-old DM patient with anti-MDA5 antibodies and typical skin signs exhibited weak sarcolemmal MxA expression in the perifascicular area. MxA was detected even in some DM patients without PFA. By contrast, anti-MDA5 antibody-positive DM had the lowest relative sensitivity of MxA expression. In our three anti-MDA5-positive DM patients, the first 42-year-old patient had significant proximal muscle weakness, typical skin lesions and ILD with normal CK levels, and had no MxA expression on their myofibres. The second patient showed only sarcolemmal immunoreactivity in the myofibres of the perifascicular area, and the third one had a mild and scattered distribution pattern of MxA-positive fibres (Figure 1A–F).

**Figure 2:**
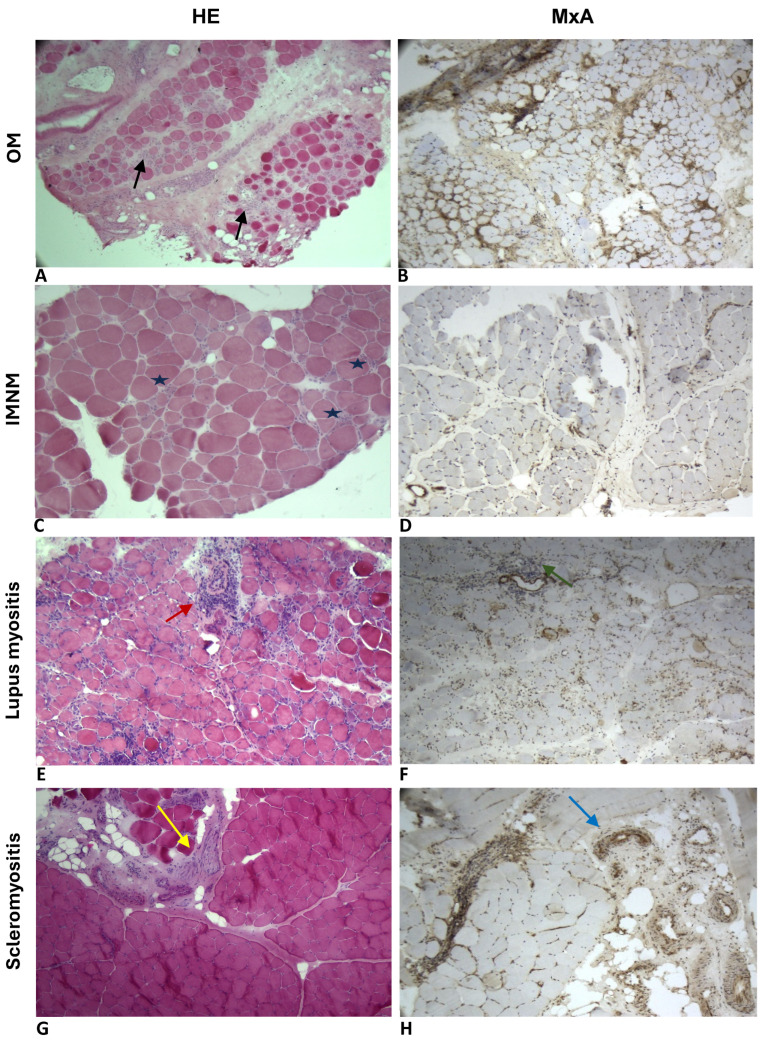
OM (**A**,**B**) with endomysial lymphocytic inflammation (black arrows) (**A**) and negative sarcoplasmic MxA expression (**B**). IMNM (**C**,**D**) with necrotizing myopathy pattern (asterix) (**C**) and negative sarcoplasmic MxA expression (**D**). Lupus myositis (**E**,**F**) with perivascular inflammatory infiltrate (red arrow) (**E**) and MxA deposition on capillaries (green arrow) with no sarcoplasmic expression (**F**). Scleromyositis (**G**,**H**) with predominant perivascular inflammatory infiltrate (yellow arrow) (**G**) and MxA-positive expression on connective tissue vessels (blue arrow) (**H**). Images (**A**,**B**,**D**–**G**): magnification 10×; images (**C**,**H**): magnification 40×. OM: overlap myositis; IMNM: immune-mediated necrotizing myopathy.

In the non-specific OM group, MxA expression was largely absent (21/23, 91%). However, two patients (9%) demonstrated sarcoplasmic MxA positivity: one with anti-Ro52 antibodies, and the other was seronegative for all MAAs. Additionally, a 61-year-old woman with SLE and concomitant positive anti-Ro52, anti-RNP and anti-Sm antibodies showed strongly expressed MxA in the perifascicular vessels (Figure 2A–H). We also identified MxA overexpression on the capillaries in a 58-year-old man with PMScl-75- and PMScl-100-positive scleromyositis (Figure 2A–H). All the patients with IBM, IMNM, and ASyS were negative for MxA. The multivariable analysis, through a multiple logistic regression, confirmed a significant correlation between positive sarcoplasmic MxA staining and the diagnosis of IIM (*p* = 0.003). The sensitivity of the sarcoplasmic expression of MxA in the myofibres of IIM patients was 21.7%, and the specificity was 100%.

#### 2.2.2. Group 2: Hereditary Muscle Diseases

**Figure 3:**
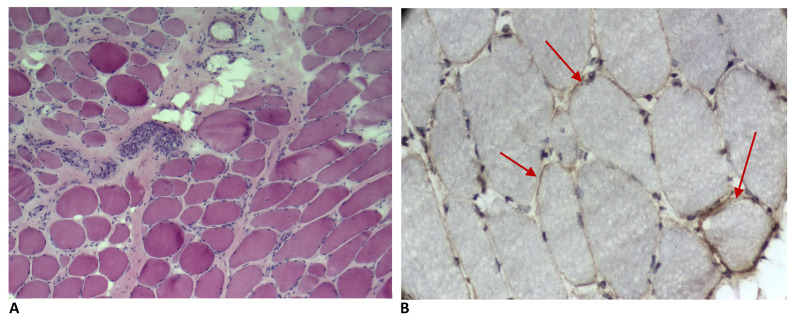
H&E staining ((**A**), magnification 40×) and MxA IHC study ((**B**), magnification 100×) showing sarcolemmal MxA expression (red arrows) in non-necrotic or regenerating fibres of patient with autosomal recessive limb-girdle muscular dystrophy.

In the HMD group, the MBs predominantly showed inflammatory cell infiltrates in addition to necrosis, regeneration and fibrosis. However, no sarcoplasmic MxA expression was observed in the non-necrotic or regenerating fibres. In contrast, a few sarcolemmal MxA-positive fibres were identified in five patients (14%): three cases of autosomal recessive LGMD, one case of distal MD and one case of CMD. Notably, no sarcolemmal MxA expression was found in the myofibres of patients diagnosed with Duchenne muscular dystrophy (Figure 3A,B).

## 3. Discussion

IIMs are rare diseases for which there is a paucity of studies in Africa [25]. Here, we highlight a series of IIM cases across the African continent and show the usefulness of MxA staining for distinguishing between IIM and HMD. Our results are consistent with those of previous studies showing that MxA expression accurately identifies patients with DM [20]. We conclude through this study that the IHC of MxA antibody could be a practical and reliable tool to differentiate JM from HMD.

The first evidence of the involvement of an IFN pathway in IIM was demonstrated in 1989 by Emslie-Smith et al. in juvenile DM [26]. Three different families of ligands activating the IFN pathway by binding to cell surface receptors are actually known: type 1 IFN (including IFN-α and IFN-β), type 2 IFN (including IFN-γ), and type 3 IFN (including IFN-λ). These proteins bind to their corresponding surface receptors, which, via the Janus kinase (JAK)/signal transducer and activator of transcription (e.g., STAT1 and-2) signalling pathways, stimulate the expression of IFN-inducible genes [27,28]. The MxA, ISG15 and ISG16 genes are specifically stimulated by IFN-I. JAK plays a key role in the expression of these genes in the target organs by phosphorylating the transcriptional factors for interferon-inducible genes, STAT1 and STAT2 [29,30]. In 2005, Greenberg et al. demonstrated that IFN-I-inducible genes were the most highly differentially expressed genes in the myofibres and endothelial cells of MBs from patients with DM [31]. IFN-Is have also been recognized to play key roles in the initiation and maintenance of the DM process through the exacerbation of inflammatory responses, upregulation of MHC-I in myofibres, their effects on endothelial cells and induction of mitochondrial dysfunction [32,33]. Additionally, plasmacytoid dendritic cells are particularly increased in DM muscle, and represent the major source of IFN-I [34]. In contrast to DM, the role of IFNs has not been well established in other types of IIM [35]. The upregulation of IFN-I-inducible transcripts is markedly and specifically increased in DM compared to other subtypes of IIM and normal muscle [18,19].

Subsequently, an increasing number of reports have noted that perifascicular atrophic fibres strongly express MxA protein in DM [20,21]. Uruha et al. reported that IHC detection of MxA protein on the sarcoplasm of myofibres is a sensitive pathological marker of DM. The sensitivity and specificity of sarcoplasmic MxA expression was 71% and 98%, respectively, revealing that it had a higher sensitivity than that of PFA and MAC deposition on capillaries (47% and 35% respectively), with an equal or higher specificity. Its diagnostic superiority over PFA was also statistically demonstrated [20]. In the 2018 ENMC workshop, MxA protein overexpression was officially included as a definitive pathologic criterion for DM classification [36]. Our study confirms the important role of MxA expression on MB samples for diagnosing DM. Sarcoplasmic MxA expression was highly prevalent in the DM subgroup compared to other IIMs, with a detected overexpression in 80% of our DM patients. A mild cytoplasmic MxA immunoreactivity in the skeletal muscle has been reported in 50 to 65% of patients with anti-MDA5 DM [21,37]. It remains unclear why anti-MDA5-positive DM exhibits a distinct morphological pattern and divergent MxA expression compared to other serological subtypes. Clinically, however, this distinction may be relevant, since anti-MDA5 DM often presents with severe extramuscular features, such as rapidly progressive interstitial lung disease, suggesting that MxA negativity in the muscle does not exclude a severe disease phenotype, as described by Englert B et al. [38]. The morphological patterns and MxA expression abnormalities in anti-MDA5-positive DM are both distinct from other serological subtypes of DM.

Interestingly, all samples of juvenile DM showed a particularly intense perifascicular MxA expression. In these cases, anti-NXP2 and anti-TIF1γ were the most frequently detected MSAs. Few studies have evaluated MxA protein expression on the myofibres of juvenile form of DM, which ranges from 61.2% up to 100% of cases [21,39]. In addition, previous studies have also showed an association between the MxA expression level and muscle disease activity in juvenile DM [40]. An assessment of the disease activity in our study was not possible due to the limited follow-up data, raising the question of the usefulness of MxA expression not only as a diagnostic marker but also as a potential biomarker of disease activity in our population.

In other types of IIM, such as ASyS, IMNM and IBM, MxA overexpression has rarely been reported [41]. The majority of ASyS myositis cases were negative for MxA sarcoplasmic expression in previous studies [20,42]. In a cohort of 194 reported ASyS cases, only three patients with anti-Jo1, anti-PL7 and anti-OJ antibodies showed myofibre MxA expression, suggesting that the IFN-I pathway may be mildly activated in a limited number of ASyS patients [43]. This was also shown at the transcriptomic level in the study by Pinal-Fernandez et al. [24]. In our study, all the ASyS patients were negative for MxA staining, suggesting a different aetiology than IFN-I activation.

Recently, MxA overexpression was reported in the myofibres of patients with SLE myositis, with statistically similar results as for those with DM [40]. Even though, pathophysiologically, SLE and scleroderma are considered IFN-I-driven autoimmune diseases [43,44], none of our five patients with active SLE myositis or scleromyositis showed MxA expression on their myofibres. This discrepancy may be explained by methodological factors. In fact, previous studies have reported that not only the sarcoplasm, but also intramuscular capillaries and other blood vessels may be highlighted by MxA immunostaining [18,40]. Nevertheless, it is important to note that other authors did not take capillary MxA staining into account because positively stained blood vessels were often seen in their samples [21]. Moreover, abnormal scattered sarcoplasmic MxA immunoreactivity was observed in 18% of sarcoidosis patients with symptomatic myopathy [45]. Finally, sarcoplasmic MxA expression was negative in reported IMNM cases, reflecting the absence of upregulation of IFN-I-inducible genes in this subgroup [40]. This is also confirmed in our study and has been reported at the transcriptomic level as well [24].

The aim of all previous studies, and this current study, have been to prove the crucial role of IFN-I in the pathophysiology of IIM and especially in DM, because specific approaches to its neutralization may constitute a promising therapeutic perspective [46]. JAK-inhibitors have been used to block the IFN-I pathway in adult and juvenile DM [47,48]. Many studies have reported the therapeutic efficacy of JAK-inhibitors such as tofacitinib and ruxolitinib in refractory DM [49]. None of our DM patients received JAK-inhibitor treatment. In contrast to IIM, the IFN-I pathway has not been established in MD, although it has become increasingly recognized that inflammation is both an early and important pathophysiological event in MD [50]. In fact, the absence of/reduction in sarcolemmal proteins causes its fragility, leading to myofibre necrosis, degeneration and regeneration cycles, and chronic inflammation [51,52,53]. There is also evidence for immunological involvement in the pathological expression of HMD, such as onset triggered by viral infections; upregulation of MHC-I in MB; and response to steroid therapy, even if transient [54,55].

On the other hand, mutations in the IFN pathways can lead to genetic diseases, such as monogenic interferonopathies [56]. STING-associated vasculopathy with onset in infancy (SAVI), a rare type 1 monogenic interferonopathy identified in 2014, caused by gain-of-function mutations in the *STING1* gene, is typically characterized by early-onset cutaneous vasculopathy, ILD, and prominent systemic features. Episodes of myositis and muscle atrophy have been reported [57]. The second genetic interferonopathy with muscle involvement is Chronic Atypical Neutrophilic Dermatosis with Lipodystrophy and Elevated temperature (CANDLE), a rare autosomal recessive IFN-I disease causing episodes of fever, skin lesions, muscle wasting, progressive lipodystrophy and joint contractures starting from the first two weeks of life [58]. Genetic type I interferonopathies share common features with DM. Muscle inflammation may be observed in 25% to 80% of patients with CANDLE and SAVI, respectively. In genetic interferonopathies, similarities with patients with anti-MDA5+ DM have been reported: ILD; non-erosive arthritis; synovitis; and severe skin lesions, such as erythematous oedema and ulcerations [57,58,59].

In the HMD group of the current study, five MD patients showed positive labelling of MxA restricted to the sarcolemmal membrane, including non-necrotic or regenerating fibres, suggesting a possible role of the IFN-I pathway in the physiopathology of those MD cases. Interpreting these relationships is difficult due to the limited number of cases. Different techniques and protocols in laboratory processes may result in different interpretations of MxA expression. In the near future, transcriptomic studies of inflammatory myopathies and genetic myopathies will likely pave the way for understanding specific interferon pathways, including MxA, and which cells express interferon-induced genes [60]. The conflicting results from different studies could partly be attributed to the use of different anti-MxA antibody products for IHC and also differences in methodology.

## 4. Materials and Methods

### 4.1. Patients

We selected 81 cases among 330 patients who underwent an MB between March 2022 and September 2024 (30 months). The patients were from different North African countries and of different races and ethnicities, mainly from Tunisia, but also from Libya, Algeria, Mauritania, Mali and Chad. The inclusion criterion was the presence of inflammatory cell infiltrate in patients’ skeletal MBs [6]. In our study, the cohort included 46 patients with IIM and 35 patients with HMD. We also included, as negative controls, 4 patients diagnosed with fibromyalgia, with either normal or neurogenic patterns on their MBs. Clinical, serological and radiological data were collected, in addition to morphological and genetic results. Information about treatment and evolution were also collected. Serum creatine kinase (CK) and electromyography were tested in all the patients. Muscle magnetic resonance imaging was performed in 17 patients (20%), among them 7 patients with HMD to determine the MB site, and 10 patients with IIM not for a diagnostic purpose.

In the IIM group (*n* = 46), anti-nuclear antibodies were assessed by indirect immunofluorescence on HEp-2 cells, a human epithelioma cell line, for the detection of both nuclear and cytoplasmic targets antigens. Line-blot immuno-assays with recombinant or native antigens were performed on all patients’ sera by the Department of Clinical Immunology at the Pasteur Institute of Tunisia according to the manufacturer’s instructions (EUROLINE Autoimmune Inflammatory Myopathies 16Ag IgG Profile (EUROIMMUN, Lübek, Germany)) [61]. This test provides a semi-quantitative evaluation of myositis-specific antibodies (MSAs) and myositis-associated antibodies (MAAs). The target antigens of MSAs include the complex nucleosome-remodelling histone deacetylase (Mi-2 α and β), the small ubiquitin-like modifier-activating enzyme (SAE), nuclear matrix protein 2 (NXP2), melanoma differentiation-associated protein 5 (MDA5), transcription intermediary factor 1 λ (TIF1λ), the signal recognition particle (SRP) and the aminoacyl–tRNA synthetases (Jo1, PL7, PL12, OJ, EJ). The MAAs include anti-Ro52 antibodies, anti-Ku, PM-Scl75 and PM-Scl 100 antibodies. Anti-3-Hydroxy-3methylglutaryl-CoenzymeA reductase antibodies (anti-HMGCR) were assessed by chemiluminescence assays (Werfen). Chest scan screening for interstitial lung disease (ILD) was performed wherever feasible, especially for patients with ASyS and OM. Screening for underlying malignancies was performed in all IIM patients. The classification criteria of the EULAR/ACR 2017 for adult and juvenile IIMs and their major subgroups were used to diagnose IIM [62]. We defined patients with IIM < 18 years as having juvenile myositis (JM).

The diagnosis and classification of HMD cases (*n* = 35) were based on the clinical presentation, CK levels, and MB morphology. The 2014 guidelines of the American Academy of Neurology were used to classify patients with MD, including LGMD and distal MD [63,64]. CMD was diagnosed based on the characteristic clinical features and evidence of a dystrophic pattern on the MB [65,66]. One case of mitochondrial myopathy was diagnosed in a patient who presented with rhabdomyolysis and ragged-red fibres on their MB [67]. IHC studies were performed for 14 patients with HMD. Among them, anti-Dystrophin2 antibodies (Novocastra Labs, Buffalo Grove, IL, USA) were applied in 7 patients with suspected Duchenne muscular dystrophy, while anti-Merosin antibodies (Novocastra Labs, Buffalo Grove, IL, USA) were used in 7 patients diagnosed with CMD. In addition, genetic analyses including either a targeted LGMD gene panel or whole-exome sequencing were conducted after the MB in 5 selected patients.

### 4.2. Muscle Biopsy Procedure and Immunohistochemistry

All the MBs were performed for diagnostic purposes, in accordance with the internationally recommended standards for MB procedures [68]. The biopsy site selection was guided by the distribution and severity of muscle weakness, along with the findings from electromyography and/or muscle imaging. The deltoid muscle was the most frequently sampled (56/81 cases), followed by the quadriceps muscle (22/81 cases). Specimens were obtained via an open surgical technique under local anaesthesia. In cases where fasciitis was clinically suspected, adjacent fascia tissue was also collected. The muscle samples were immediately snap-frozen in isopentane cooled in liquid nitrogen and stored at −40 °C in an ultra-low temperature freezer until further processing. The frozen muscle sections were prepared using a cryostat (Leica Biosystems, Victoria, Australia) and stained using standard histological and histochemical techniques. These included haematoxylin and eosin (H&E)-modified Gomori trichrome, and a panel of enzymatic stains: nicotinamide adenine dinucleotide–tetrazolium reductase, succinic dehydrogenase, periodic acid–Schiff, and Sudan Black.

For the IHC, serial 7 μm cryostat sections were mounted on gelatine-coated glass slides at −20 °C, air-dried at room temperature for 30 min, and stained for MxA. The primary antibody used was a polyclonal anti-human MxA antibody (Ab95926; dilution 1:200; Abcam, Waltham, MA, USA). Staining was performed using a fully automated BOND-MAX IHC system (Leica Biosystems, Victoria, Australia). All the slides were dehydrated in 100% ethanol (twice), cleared in xylene (twice), and mounted with coverslips. All the IHC procedures were conducted in the same pathology laboratory under standardized conditions.

### 4.3. Histological and Immunohistochemical Evaluation

The histological evaluation was performed using a semi-quantitative scoring system assessing four domains: muscle fibre morphology, inflammatory infiltrate, vascular changes, and connective tissue alterations. This system was adapted from previously published juvenile DM pathology scoring methods [69]. Inflammatory infiltration was graded as: (−) absent, (+) mild, (++) moderate, or (+++) marked.

For the IHC evaluation, positive MxA expression in myofibres was defined as either sarcoplasmic (within the myofibre cytoplasm) or sarcolemmal (membrane) staining, excluding necrotic and regenerating fibres, as described previously [20]. The sarcoplasmic staining intensity was graded as follows: (−) absent, (+) weak, (++) moderate, or (+++) strong. The sarcolemmal staining was reported as either present or absent.

This method is different from those used routinely in the assessment of other IHC markers of inflammatory myopathies, such as MHC-I and MAC. For example, the evaluation of the expression of MHC-I is mainly based on the sarcolemmal expression intensity [70].

The distribution pattern of MxA expression was also recorded and categorized into one of three types:

1. Perifascicular: Restricted to perifascicular fibres.
2. Scattered: Irregularly distributed without localization.
3. Diffuse: Widespread expression across the biopsy.

**Figure 4:**
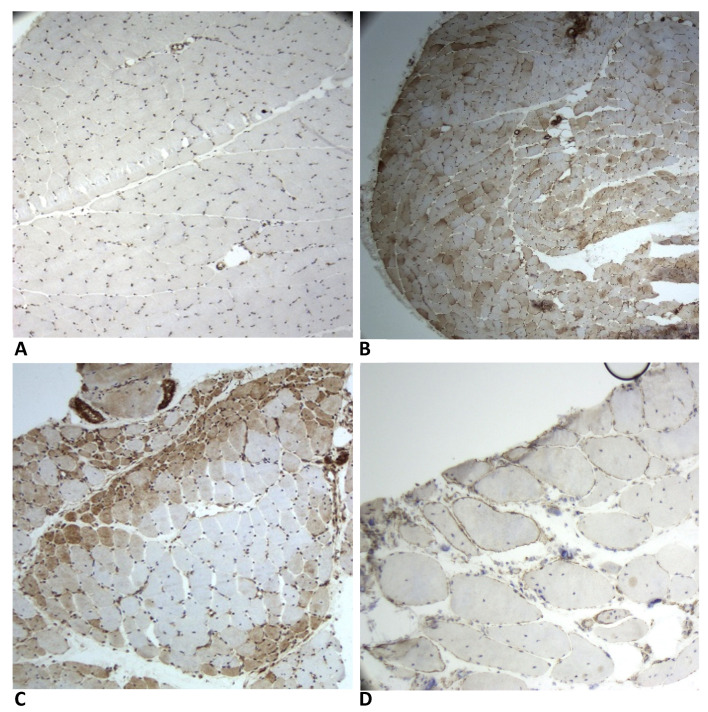
Representative immunohistochemical staining of MxA in muscle biopsies of patients with different diagnoses: (**A**) negative control, (**B**) anti-NXP2-positive DM, (**C**) seronegative juvenile DM, and (**D**) CMD. The sarcoplasmic intensity is categorized into one of four graduations: absent (**A**), poorly stained (**B**), moderate (**C**), or marked. Three patterns of MxA-positive fibres distributions were recognized: perifascicular (**C**), diffuse (**B**) and scattered. Sarcolemmal-positive staining is retained in non-necrotic or regenerating fibres (**D**). Images (**A**,**B**): magnification 10×; image (**C**): magnification 40×; image (**D**): magnification 100×.

The representative staining patterns are illustrated in Figure 4A–D.

### 4.4. Ethical Approval and Patient Consent

The study was approved by the ethics committee of the National Institute Mongi Ben Hamida of Neurology of Tunisia (Number CE 2025/20). Written informed consent was obtained from each participant.

### 4.5. Data Analysis and Statistics

The statistical analysis was carried out using IBM-SPSS software, version 27. The difference in the sarcoplasmic MxA expression between the two groups (IIM and HMD) was tested by Fisher’s exact test. Values of *p* < 0.05 were considered significant.

## 5. Conclusions

This study presents additional data on the importance of MxA use in distinguishing between IIM and HMD, and its value in the diagnosis of adult and juvenile DM. Our study includes a large cohort of patients from the North African region and compares their histological expression levels. Although there are significant differences in the types and patterns of MxA immunoreactivity between the two main groups, we demonstrate that IFN-I may be expressed in the sarcolemma of a limited number of fibres in some MD cases, suggesting the possible immunological involvement of the IFN-I pathway in MD.

This study does have several limitations. First, the small size of each group, due to the low prevalence of myopathies, constrained the overall sample size and statistical power of the study. Moreover, the IIM group included patients with heterogeneous disease activity levels and some of them were on treatments at the time of biopsy. Second, a major segment of the HMD patients had not received a precise molecular diagnosis due to the limited access to genetic labs in the region. Finally, a single reader performed the determination of MxA staining patterns, lacking an inter- and intrareader variability assessment, as this study was conducted in the first North African centre performing fully automated IHC of the IIM markers of muscle tissue. We hope that further studies analysing IFN signatures could help in the comprehension of how the immune system is involved in IIM and also in HMD, which may have implications for future therapeutic approaches.

## Figures and Tables

**Table 1:** Demographic, clinical, and serological features of IIM group.

	DM	IBM	IMNM	ASyS	Non-Specific OM	Scleromyositis
Age at biopsy (years)	44 (11–73)	56.5 (56–57)	60 (48–75)	56 (45–68)	42.5 (8–78)	50 (58–42)
Adult (≥18 years old)	7 (70%)	2	5	4	20 (87%)	2
Juvenile (<18 years old)	3 (30%)	0	0	0	3 (13%)	0
Female/male	9/10	1/1	3/2	3/1	19/4	1/1
Disease duration before biopsy (months)	6, 8 (1–36)	12	6, 6 (3–18)	38 (24–67)	14 (1–96)	9 (6–12)
Comorbidities						
Hypertension	1 (10%)	0%	1 (20%)	0%	5 (22%)	0%
Diabetes	1 (10%)	0%	3 (60%)	0%	6 (26%)	0%
Dyslipidaemia	0%	0%	3 (60%)	0%	1 (4%)	0%
Thyroid diseases	0%	0%	0%	0%	4 (17%)	1 (50%)
Autoimmune diseases	1 (10%)	1 (50%)	0%	2 (50%)	7 (30%)	0%
Clinical manifestations						
Muscle weakness	9 (90%)	2 (100%)	5 (100%)	4 (100%)	17 (74%)	2 (100%)
Swallowing difficulties	4 (40%)	0%	1 (20%)	2 (50%)	8 (35%)	1 (50%)
Respiratory difficulties	2 (20%)	0%	1 (20%)	2 (50%)	4 (17%)	1 (50%)
Extramuscular manifestations						
Skin lesions	9 (90%)	0%	0%	2 (50%)	8 (35%)	2 (100%)
Joint involvement	7 (70%)	0%	0%	2 (50%)	16 (48%)	1 (50%)
ILD	2 (20%)	0%	0%	3 (75%)	0%	1 (50%)
Raynaud phenomenon	1 (10%)	0%	0%	0%	0%	0%
CK (IU/L)	1063 (200–5000)	340 (200–480)	2430 (1000–4520)	347.5 (200–790)	980 (200–7000)	200

CK: Serum creatine kinase; ILD: interstitial lung disease; DM: dermatomyositis; IBM: inclusion body myositis; IMNM: immune-mediated necrotizing myopathy; ASyS: antisynthetase syndrome; OM: overlap myositis.

**Table 2:** Demographic data and clinical features of the HMD group.

Characteristics	Median and/or Percentage
Female/male	16/19
Age at muscle biopsy (years)	20 (1.5–57)
Adult (≥18 years old)	14 (40%)
Juvenile (<18 years old)	21 (60%)
Disease duration before biopsy (months)	74 (6–200)
Family history (parental consanguinity and/or similar cases)	21 (60%)
CK (IU/L)	5320 (200–22,500)
HMD subtype, *n* (%)	
Unclassified autosomal recessive LGMD	42% (*n* = 15)
Duchenne muscular dystrophy	20% (*n* = 7)
Congenital muscular dystrophy	20% (*n* = 7)
Gamma-sarcoglycanopathy (LGMDR5)	6% (*n* = 2)
Unclassified distal muscular dystrophy	6% (*n* = 2)
Calpainopathy (LGMDR1)	3% (*n* = 1)
Metabolic myopathy	3% (*n* = 1)

HMD: hereditary muscle diseases; CK: serum creatine kinase; LGMD: limb-girdle muscular dystrophy.

**Table 3:** MxA staining in AIM and HMD patients.

	AIMs (*n* = 46)	HMDs (*n* = 35)	*p*-Value
Negative	35 (76.1%)	30 (85.7%)	Ref *
Sarcoplasmic	10 (21.7%)	0	0.003
Sarcolemmal	1 (2.2%)	5 (14.3%)	0.035
Fisher’s exact test global *p*-value = 0.001			

AIMs: autoimmune inflammatory myopathies; HMDs: hereditary muscle diseases. * Reference category.

## Data Availability

The original contributions presented in this study are included in the article. Further inquiries can be directed to the corresponding author.

## Abbreviations

The following abbreviations are used in this manuscript:

ACR	American College of Rheumatology
Anti-HMGCR	Anti-3-hydroxy-3-methylglutaryl–coenzyme A reductase
Anti-MDA5	Anti-melanoma differentiation-associated gene 5
Anti-Mi2	Anti-complex nucleosome-remodelling histone deacetylase
Anti-NXP2	Anti-nuclear matrix protein 2
Anti-SAE	Anti-small ubiquitin-like modifier-activating enzyme
Anti-SRP	Anti-signal recognition particle
Anti-TIF1γ	Anti-transcription intermediary factor 1 gamma
ASyS	Antisynthetase syndrome
CANDLE	Chronic atypical neutrophilic dermatosis with lipodystrophy and elevated temperature
CK	Serum creatine kinase
CMDs	Congenital muscular dystrophies
DM	Dermatomyositis
ENMC	European Neuromuscular Centres
EULAR	European League Against Rheumatism
H&E	Haematoxylin–eosin
HMDs	Hereditary muscle diseases
IBM	Inclusion body myositis
IIMs	Idiopathic inflammatory myopathies
IFN-I	Type I interferon
IHC	Immunohistochemistry
ILD	Interstitial lung disease
IMNM	Immune-mediated necrotizing myopathy
ISG15	Interferon-stimulated gene 15
JAK	Janus kinase
JM	Juvenile myositis
LGMDs	Limb-girdle muscular dystrophies
MAC	Membrane attack complex
MAAs	Myositis-associated antibodies
MB	Muscle biopsy
MDs	Muscular dystrophies
MHC-I	Major histocompatibility complex class I
MSAs	Muscle-specific antibodies
MxA	Myxovirus resistance A
OM	Overlap myositis
PFA	Perifascicular atrophy
SAVI	STING-associated vasculopathy with onset in infancy
SLE	Systemic lupus erythematosus
STAT	Signal transducer and activator of transcription
